# Management of Ellis Class-IV Fractured Tooth With an Open Apex: A Case Report

**DOI:** 10.7759/cureus.29681

**Published:** 2022-09-28

**Authors:** Radhika Gupta, Aditya Patel, Pradnya P Nikhade, Manoj Chandak, Anuja Ikhar

**Affiliations:** 1 Department of Conservative Dentistry and Endodontics, Sharad Pawar Dental College, Datta Meghe Institute of Medical Sciences, Wardha, IND

**Keywords:** glass fiber post, immature tooth, open apex, ellis class iv fracture, mta apexification

## Abstract

Dental professionals face difficulty in managing "immature non-vital teeth with an open apex." It is considered to be challenging because, in this situation, root canal filling material should be confined within the boundaries of the root canal without extruding peri-apically. Apexification tries to create a hard tissue barrier that will cause the open apex to close, allowing root filling to be compacted. The current case report describes the application of mineral trioxide aggregate (MTA) to create an apical plug in an open apex, as MTA is considered a versatile material for apexification, which was followed by the reinforcing of the weekend root using glass fiber post and core build-up to manage the Ellis class IV fracture.

## Introduction

The majority of facial injuries frequently involve traumatic dental injuries. Although traumatic injuries can happen to anyone at any age, children account for 30% of all traumatic dental injuries [1]. The majority of these injuries happen before the root development has been completed [2]. An immature apex with weak dentin walls and an early loss of vital pulp leaves the tooth vulnerable with a compromised ratio of the crown to the root [3]. In young children, traumatic dental injuries can lead to pulpal necrosis and may terminate the continuation of further root development, leading to the development of open apices. Another cause of an open apex is severe resorption of a mature apex after orthodontic therapy or peri-radicular inflammation [4].

Non-blunderbuss and blunderbuss are two types of open apices. A non-blunderbuss is a type of open apex where the walls are parallel to slightly convergent as the canal exits the root and the apex is mostly convergent. Another one is the blunderbuss, in which walls are divergent and flaring, especially in the buccolingual direction, with a funnel-shaped apex that is wider than the coronal aspect. Incomplete development, pulp necrosis induced by caries or trauma before the root formation has been completed, extensive apical resorption, root end surgery, and aggressive instrumentation are the major causes of open apices [5].

Managing the case of the open apex is challenging for the clinician because of the difficulty in debriding, cleaning, and obtaining appropriate sealing of the root canal space. As the apex has not fully developed, there is no barrier to prevent the root canal filling material from entering and damaging the peri-apical tissues. Fractures of the root can occur during and after treatment because of thin dentinal walls. These complications are resolved by encouraging the formation of a calcific barrier to enable adequate root canal filling. This calcific barrier also helps in strengthening the weak root structure [1].

Apexification is an endodontic procedure involving placing a calcific barrier over an exposed tooth's apex to achieve apical healing. The procedure typically treats permanent teeth with an open apex and non-vital pulp. Long-term endodontic effectiveness depends on thoroughly disinfecting the root canal system by cleaning and shaping, followed by homogenous obturation. Lack of natural apical constriction is a problem in some situations, such as with developing teeth. One of the goals of endodontic therapy is to create an apical stop or barrier so that one can place root canal filling material against it without excessive extrusion [6].

Around the world, different materials are employed in various techniques to stimulate the formation of the root end barrier. Materials that have been utilized in the past to manage teeth with open apexes include "calcium hydroxide, freeze-dried allogenic dentin powder, bone ceramic, tricalcium phosphate, osteogenic protein, collagen, calcium gel, and most recently mineral trioxide aggregate (MTA) and Biodentine" [7]. MTA has been demonstrated to be a very effective root-filling material for sealing immature root canals with open apices that could present technical issues in achieving proper obturation. MTA can promote the creation of hard tissue, which aids in peri-radicular healing [8]. Importantly, MTA shortens appointment times and potentially permits apexification in a single visit. The MTA provides a reliable apical barrier [8].

In this case, the fracture line is extending in an oblique pattern towards the cervical area but no mobile fracture fragment is seen. To rehabilitate the intraarticular portion of the tooth, a post is used, which provides enough strength to withstand fracture due to undue forces. Therefore, the current case report emphasizes the "Nonsurgical management of asymptomatic teeth with open apex with periapical radiolucency using MTA for apexification and glass fiber post" to rehabilitate the intra-radicular portion of the fractured tooth, followed by composite core build-up and prosthesis.

## Case presentation

**Figure 1:**
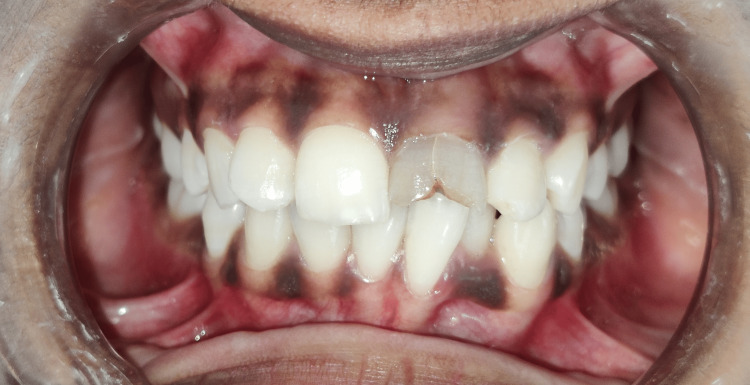
Pre-operative clinical picture of Ellis Class IV fracture with 21

In the "Department of Conservative Dentistry and Endodontics, Sharad Pawar Dental College, Sawangi," a 19-year-old female patient came with a chief complaint of a discolored and fractured tooth in the upper left front region of her jaw. The history of trauma around nine years ago due to a fall was given by the patient. The patient presented with no swelling or pain. The clinical investigation showed a discolored tooth with an Ellis Class IV fracture with 21 where the fracture line extends in the cervical region involving the pulp chamber without mobility (Figure 1).

**Figure 2:**
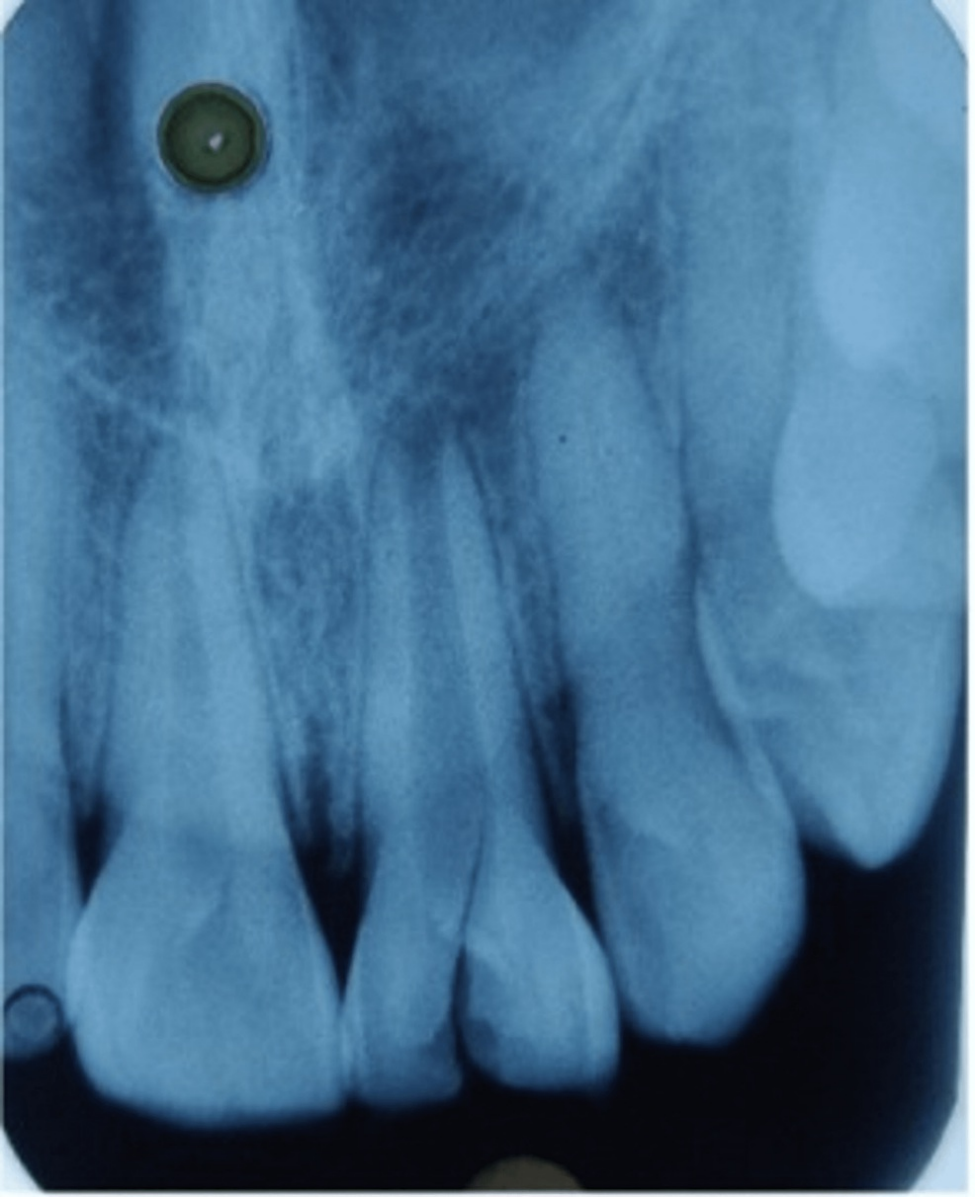
Pre-operative radiograph showing wide open apex and fracture line extension in the cervical region with 21

No response was seen with 21 by the neural sensibility (electronic pulp tester) test. The radiographic investigation presented an "immature tooth with a wide-open apex with mild radiolucency" around the periapical region (Figure 2). Hence, the final diagnosis for this case was "pulpal necrosis with asymptomatic apical periodontitis with 21."

Treatment

**Figure 3:**
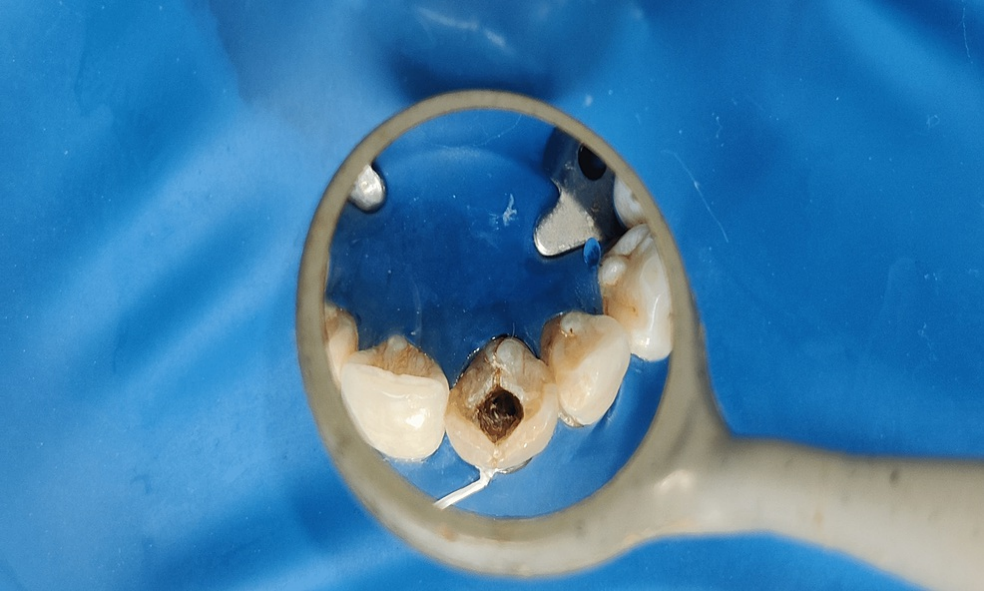
Access opening under rubber dam isolation with 21

Post and core placement for intraarticular rehabilitation of the tooth and crown prosthesis were designed after apexification using White ProRoot MTA (Maillfer, Dentsply, Switzerland). Formal consent was obtained after describing the treatment strategy to the patient. Access opening was carried out on the initial visit utilizing an "endo access bur #1 (Dentsply, Pennsylvania, US)" under rubber dam (GDC) isolation (Figure 3).

**Figure 4:**
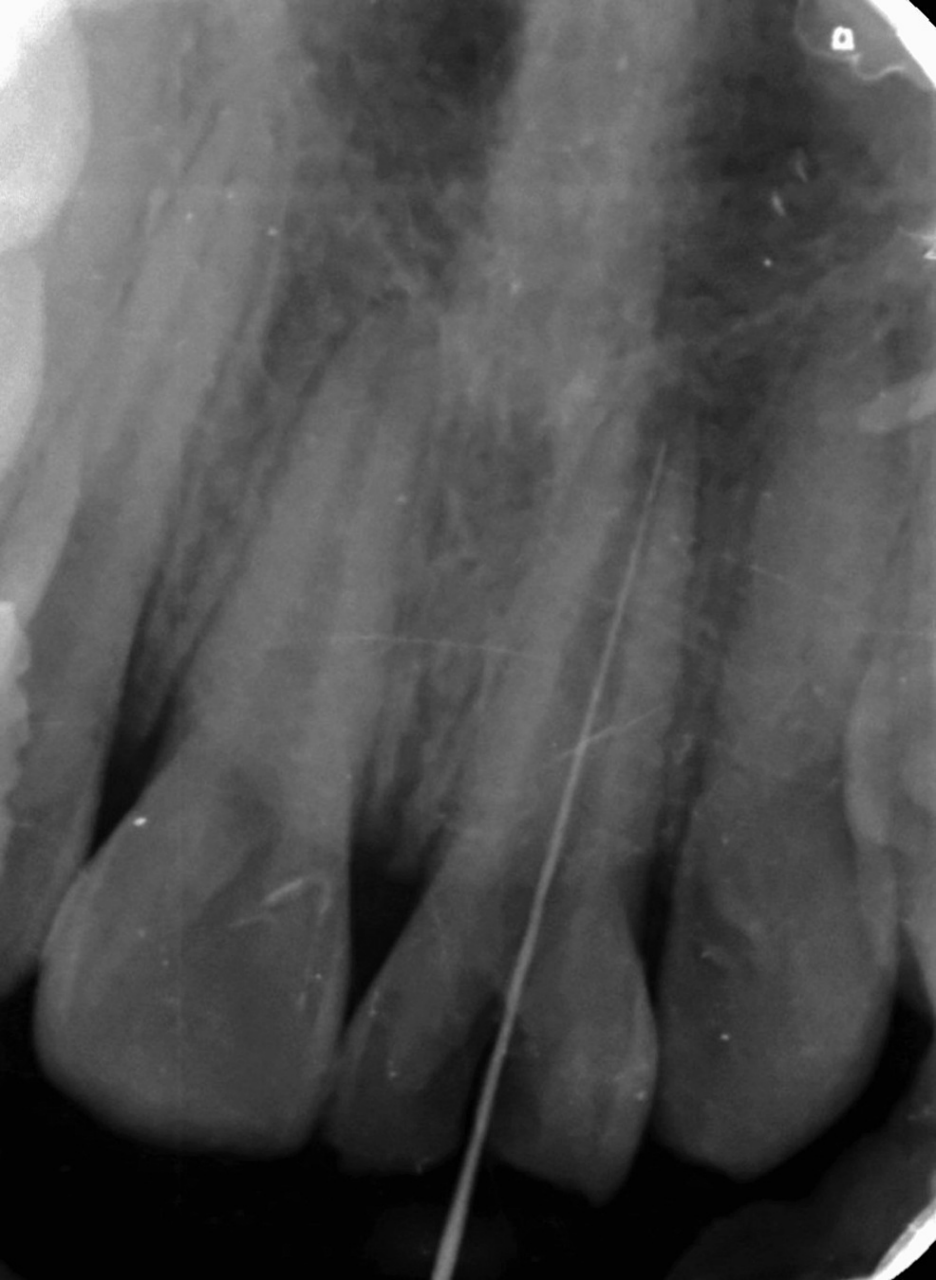
Working length determination with 21 using radiovisiography

"Using a #10 K file (Mani, Brussels, Belgium)," determination of working length was done and was verified utilizing radiovisiography (RVG) (Figure 4).

Using the step-back technique, biomechanical preparation was carried out using endodontic files ranging from #21 to #80 (Mani). 5.25% sodium hypochlorite (NaOCl) (Prime Dental Products Pvt. Ltd., India) and 0.9% normal saline were used for irrigation (Polyamp DuoFit, South Wales, Australia). As a final irrigant, 2% chlorhexidine gluconate (AnabondAsep-RC, Chennai, India) was used. The Lentulo spiral (Mani, Brussels, Belgium) was used for the application of calcium hydroxide (Prime dental RC Cal), and the tooth was then temporarily sealed and the patient was recalled after a week.

**Figure 5:**
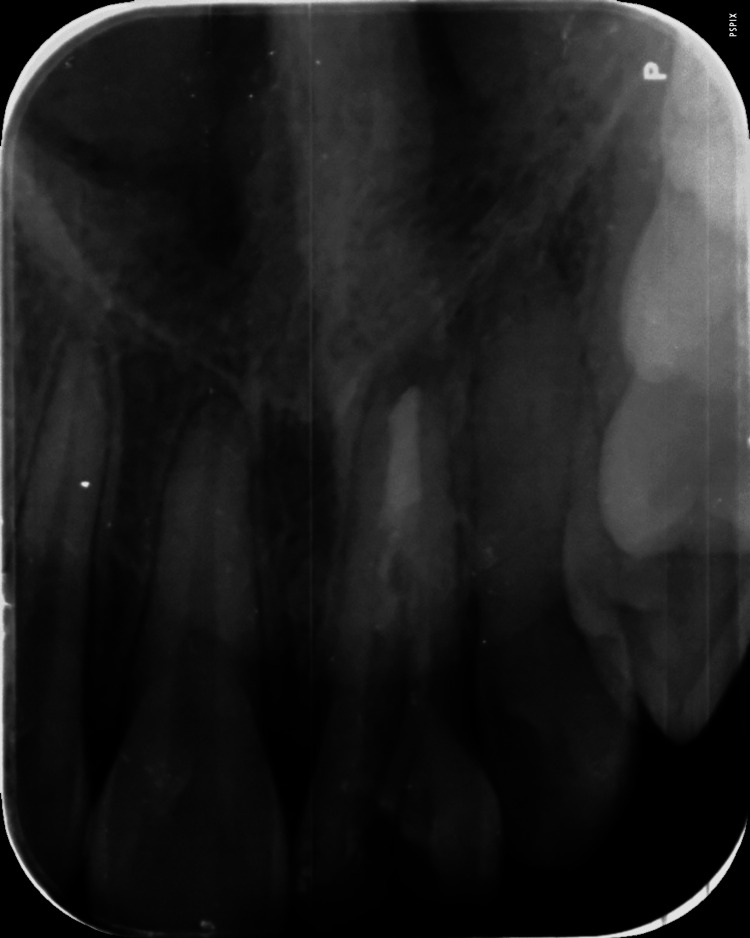
Mineral trioxide aggregate apical plug of 5 mm with 21

The access cavity was re-established on the second visit, and it was thoroughly irrigated following the same protocol and dried with sterile paper points. Manipulation of White ProRoot MTA was done according to the manufacturer's instructions and manually packed to an apical third thickness of 5 mm using a "hand plugger" (Mani, Brussels, Belgium). The placement of MTA was confirmed with RVG (Figure 5).

**Figure 6:**
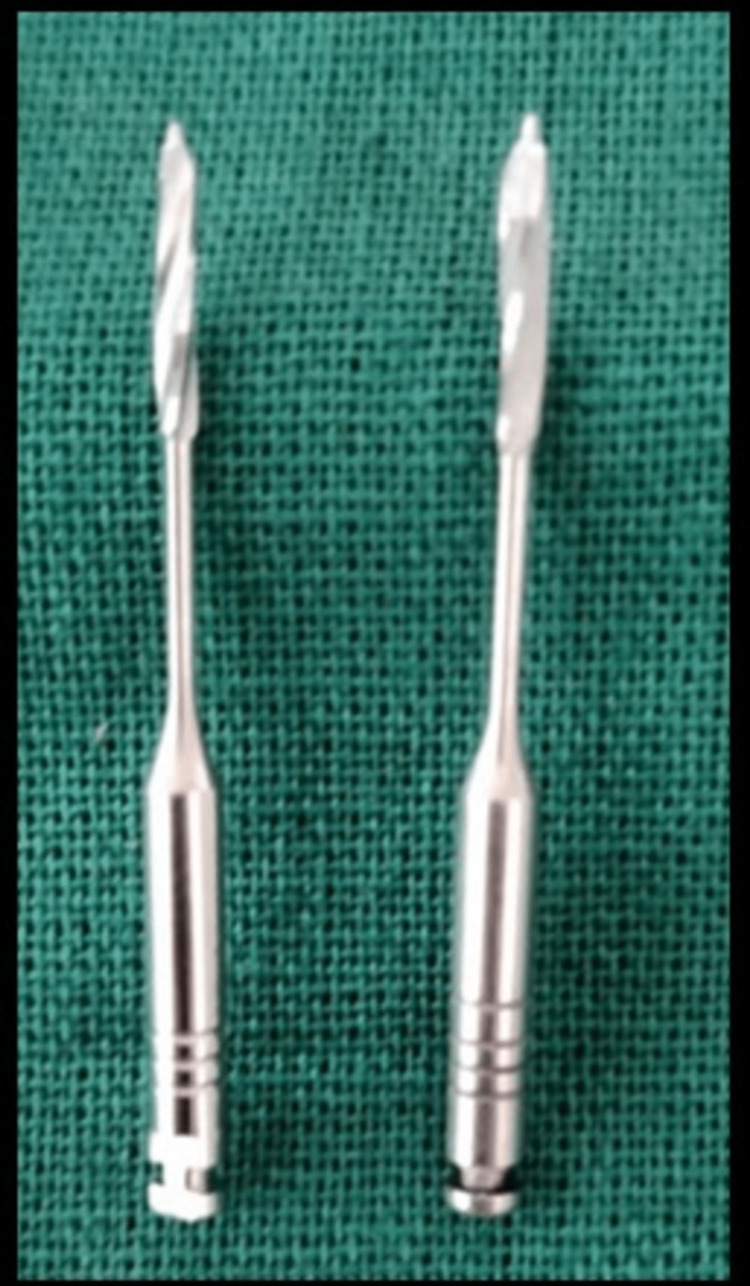
Peeso reamers nos. 3 and 4 to prepare post space

A sterile pellet of cotton was introduced into the root canal, and Cavit, a temporary filling material, was utilized to temporize the tooth. At the appointment, after two weeks, the restoration of the tooth began with the post-space preparation to rehabilitation of the intra-radicular area of a fractured anterior tooth the following visit. To prepare the post space and eliminate any potential undercut on the canal walls, Peeso reamers nos. 4 and 5 were used (Figure 6).

**Figure 7:**
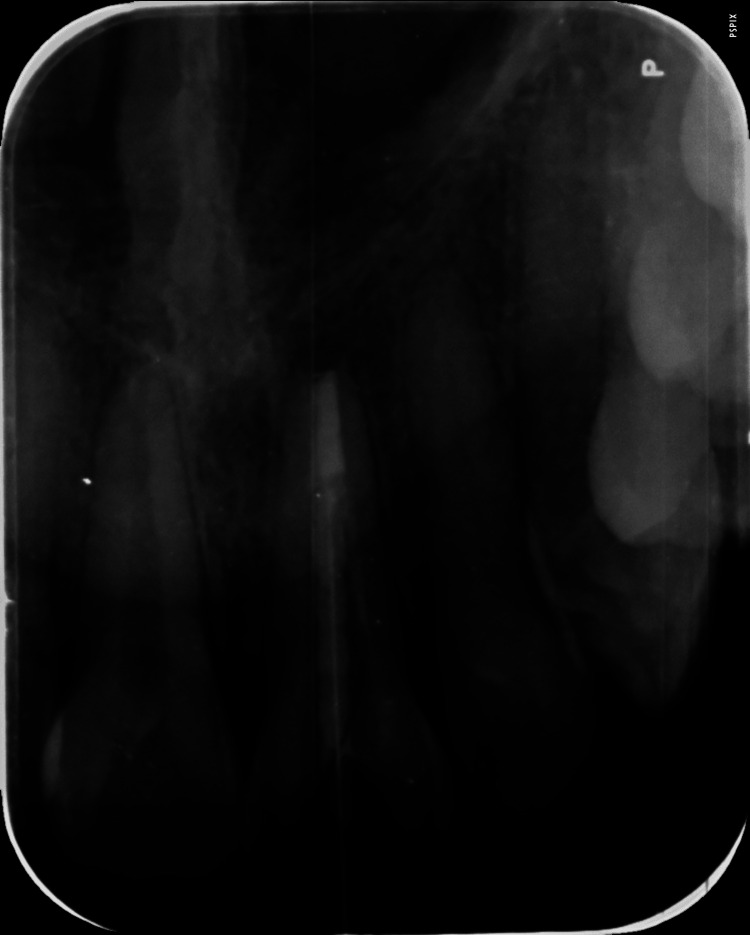
Radiographic evaluation of post fit with 21

The canal was completely air dried using a three-way syringe. A "glass fiber post" was selected (Maillefer, France), and then radiographically post fit was examined (Figure 7).

A bonding agent in the form of the Self Cure Activator self-cure bonding system using a micro brush tip was vigorously applied on the glass fiber post and then it was light cured with a light emitting diode curing device (LED) for 30 seconds. It was cured from all sides to enable the bonding between resin composites and glass fiber posts, followed by etching of the root canal wall using 37% phosphoric acid for 15 seconds, followed by water rinsing and air drying gently. Then, using the micro brush tip, the bonding agent (3M ESPE, St. Paul, USA) was applied to the root canal wall and light cured for 20 seconds, followed by glass fiber post cementation using dual cure resins, which consist of two paste systems, catalyst paste and base paste. These pastes are dispensed in a 1:1 ratio onto a clean mixing pad, then the cement is manipulated for 20-30 seconds. This manipulated paste was then coated evenly over the surface of the glass fiber post and in post space, followed by light curing for 30 seconds.

**Figure 8:**
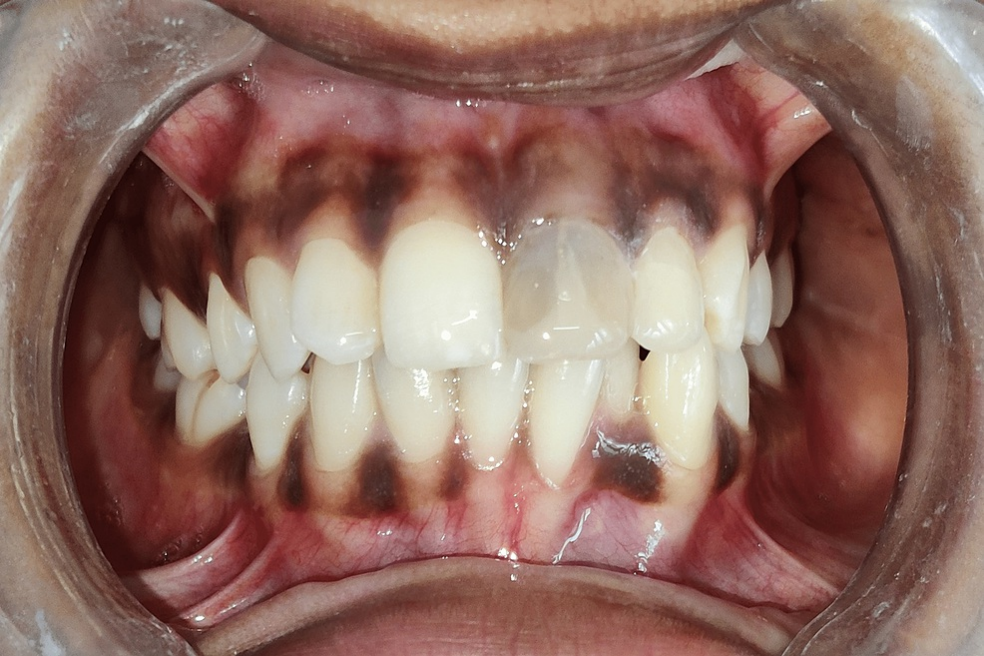
Composite core build-up with 21

Shade selection and composite core build-up were done after post-placement (Figure 8).

**Figure 9:**
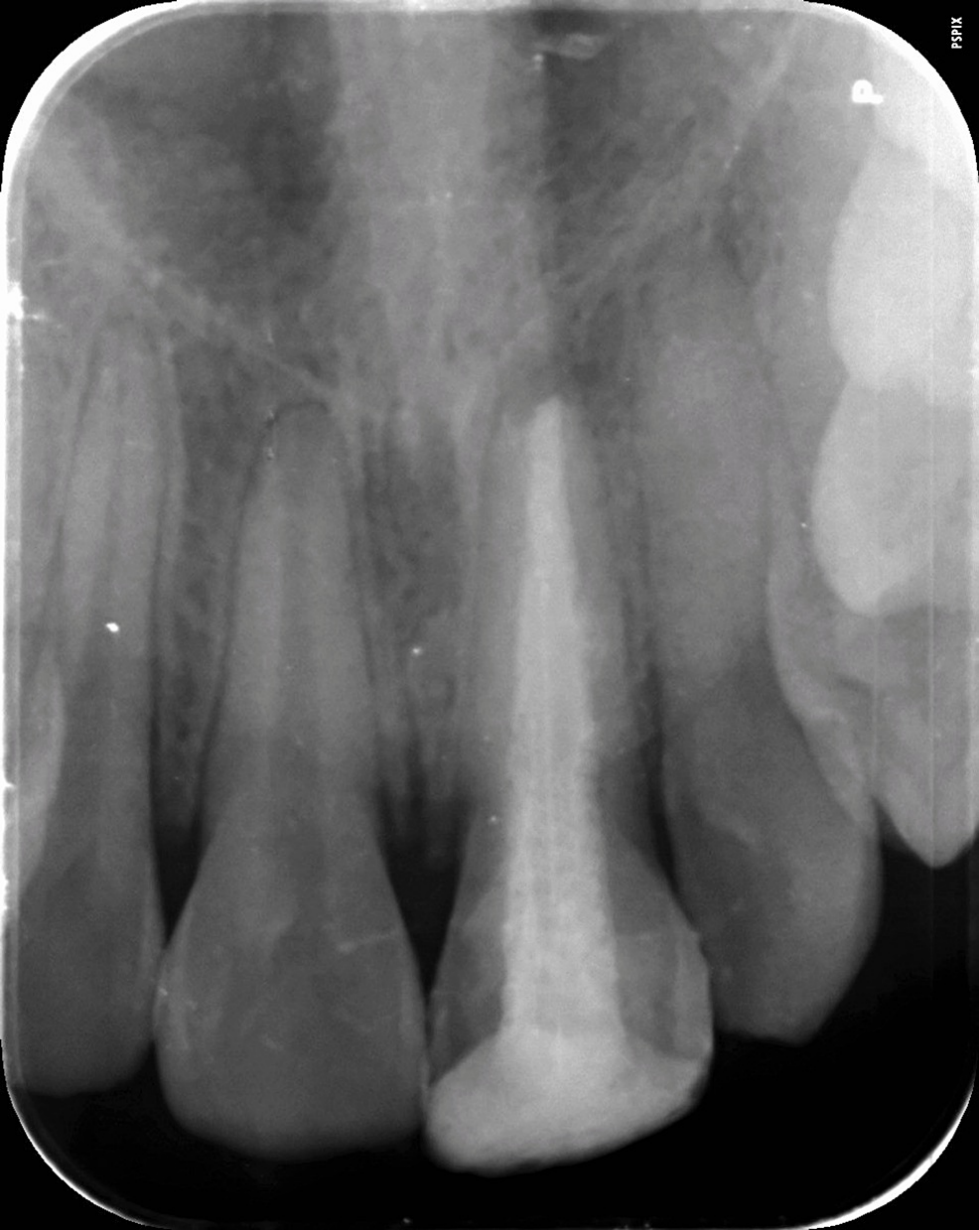
Radiographic picture of post cementation and core build-up with 21

To check the cementation of the post and core build-up, a final radiograph was taken (Figure 9).

**Figure 10:**
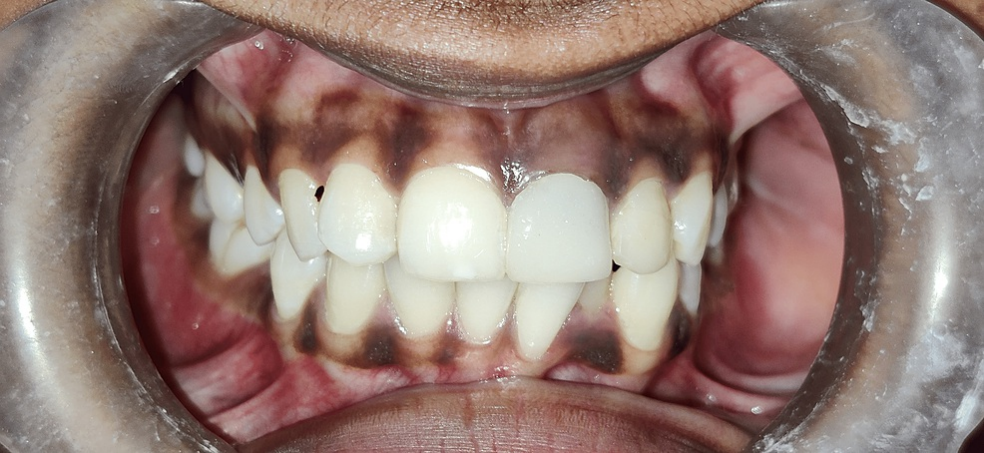
Porcelain-fused metal crown prosthesis with 21

The crown prosthesis of porcelain fused to metal was then cemented after one week of core build-up with 21 (Figure 10). The patient was routinely kept on follow-up to evaluate periapical healing.

## Discussion

In both the primary and permanent dentition, the maxillary incisors are the teeth that most frequently tend to fracture. Due to their participation in contact sports, teenagers account for a major portion of tooth injuries [9]. A non-vital tooth with or without loss of crown structure due to trauma is referred to as an Ellis class IV fracture. Severe dental injury increases the chance for micro-organisms to enter dentinal tubules and increases the risk of developing acute pulpal inflammation [10]. The open cavity in this scenario results in greater dentin demineralization because it has remained untreated for a very long period of time. When the root canal was being cleaned and shaped, more of the infected soft dentin was removed.

The requirement to limit the material to prevent excessive material from being extruded into the periodontal tissue is the main challenge in cases of a wide-open apex. Utilizing a matrix prevents material from being forced into periodontal tissues, minimizes sealing material permeability, and promotes a positive reaction in the tissues [11]. The creation of the apical barrier during apexification has utilized a variety of materials. The material of choice for establishing the apical calcific barrier has been calcium hydroxide. However, calcium hydroxide's application is constrained by how long it takes to induce apical end closure. Additionally, prolonged use of calcium hydroxide makes the wall of the root canal more brittle. MTA might be a good substitute to carry out this process with predictable outcomes. By forming a hard-tissue barrier, MTA can help to maintain proper peri-radicular architecture [12]. Its superior biocompatibility, capacity to solidify in the presence of blood, and potential for rapid tooth restoration without affecting the mechanical qualities of dentine make MTA a superior material for apexification [12-14]. To facilitate intra-radicular rehabilitation of fractured roots by using the cast post, the wall of the root canal was considerably thinned. Therefore, in this case report, the glass fiber post was selected because it offers pleasing aesthetics with its modulus of elasticity similar to that of dentin [1].

The efficiency of MTA utilized in a variety of dental treatments, including apexification, has been confirmed by many investigators [15-18]. In addition, Muhamad et al. concluded that MTA can stimulate the formation of bone and cementum and also cause less inflammation as compared to other materials [19]. According to studies on human osteoblasts, MTA promotes the secretion of cytokines by promoting the growth of bone cells, and cytokines regulate bone metabolism. These findings showed that MTA may be used to encourage the creation of hard tissues.

Some authors found that there is reduced bacterial leakage with the MTA seal as compared to SuperEBA, IRM, and amalgam after examining the MTA seal (microleakage) [7,19,20]. Tolibah et al. conducted a "Randomized Clinical Trial comparing the efficiency of MTA versus biodentin in the apexification procedure for immature nonvital first permanent molars." In this study, they concluded that biodentin can be used as an apical plug to treat immature permanent molars with periapical lesions. It also showed favorable outcomes in terms of healing of the periapical area, which was comparable with MTA, and also that biodentin has decreased treatment time [21]. Before using MTA, Torabinejad and Chivian advise using calcium hydroxide for the first week to control bleeding from the periapical region. Additionally, the authors of this investigation used a calcium hydroxide disinfection insert [22]. Using calcium hydroxide as an intracanal medicament and MTA, followed by placement of glass fiber post and core, Torabinejad et al. [23] have described a case of successful treatment of an open apex case of a badly broken upper central incisor.

After the placement of 5 mm of the MTA apical plug and the superficial pre-treatment of the glass fiber post, the reinforcement of the fiber post was done with composite resin to accommodate the remaining areas around the wall of the root canal. To evenly deliver the forces to the root canal, the entire glass fiber post with its luting cement functions as a "single unit," which depicts the concept of "Monoblock." Glass fiber posts are superior to traditional cast metal posts in that their reduced elastic modulus shields them from root fracture by minimizing stress spread from the post to the root. The wedging effect of a cast metal post would cause a root fracture [24].

The conventional custom-cast dowel core nearly always requires the least amount of tooth structure removal and offers improved geometric adaptability to extremely broad or elliptical root canals. In conditions where the coronal tooth structure is absent or present but in small amounts, tapered root canals and canals with irregular cross-sections, custom cast posts, and cores are the best choices of treatment. This method combines the benefits of fiber posts with specially designed posts [25]. The material which is used to restore endodontically treated teeth always should have mechanical properties similar to the tooth structure which it is replacing, to support this glass fiber post was introduced, which has similar mechanical properties as well as is highly aesthetic [26]. Since fiber posts have a favorable matching elastic modulus (30-40 GPa) to dentin (15-25 GPa), it creates a homogenized unit in the root canal system. After conditioning and bonding treatments, composite resin adheres well to the dentinal walls and helps to strengthen the weak root. The elastic modulus of composite resin is close to that of dentin, which is 20 GPa to 18 GPa. This is responsible for the enhancement of mechanical properties. This results in a more uniform distribution of forces on the root and reduces the chances of a root fracture [9]. Porcelain-fused metal crowns are used for post-endodontic prostheses because of their high translucency and conservative preparation design, which better meets the aesthetic needs of the patient.

The present case illustrates that the one-visit apexification method can be used in immature, non-vital teeth with or without peri-radicular infection. It should be kept in mind that the outcome and timing of closing the apex depend on several factors, including the type of material utilized, the treatment process, the correct diagnosis, the instrumentation technique, and the root canal disinfection. The patient's general health is also important since it affects his ability to regenerate periodontal structures. The authors concluded that, based on research results to date, MTA might be employed in apexification with excellent results [15].

## Conclusions

To form the calcific barrier in the open apex case and to prevent the extrusion of the obturating material in the periapical area, MTA is considered the best material. It produces a strong periapical seal and allows the creation of a post that is strong enough. Following acid etching and tooth bonding, composite resin adheres effectively to the dentinal wall and helps to strengthen the weak root structure. Complete polymerization to the depths of the canal is made possible by the use of "fiber posts and dual-cure resin." The positioning of the "fiber post" and the construction of the composite resin core guarantee the best resistance and retention form. Additionally, it has greater aesthetics. Therefore, even severely damaged teeth do not always need to be extracted. To best meet the patient's needs, they can be restored using this method.
